# Prevalence of Fetal Alcohol Exposure by Analysis of Meconium Fatty Acid Ethyl Esters; A National Canadian Study

**DOI:** 10.1038/s41598-019-38856-5

**Published:** 2019-02-19

**Authors:** Kaitlyn Delano, Gideon Koren, Martin Zack, Bhushan M. Kapur

**Affiliations:** 1 The Departments of Pharmaceutical Sciences University of Toronto, Toronto, Canada; 2 Motherisk Israel and Maccabi- Kahn Institute of Research and Innovation, Tel Aviv, Israel; 3 Tel Aviv University, Tel Aviv, Israel; 4 University of Western Ontario, London, Canada; 5 Department of laboratory medicine, University of Toronto, Toronto, Canada

## Abstract

Our study aimed to estimate the prevalence of heavy fetal alcohol exposure through the analysis of meconium FAEEs as an objective biomarker of fetal exposure. We conducted a study on meconium samples collected nationwide through the Maternal-Infant Research on Environmental Chemicals (MIREC) Study Group. FAEE in meconium was quantified by an established headspace solid-phase microextraction coupled with gas chromatography-mass spectrometry (SPME GC-MS). Out of 1315 samples collected in 10 Canadian obstetric units coast to coast between 2008-2011, the estimated prevalence of positive meconium FAEE ranged between 1.16% and 2.40%, translating into at least 1800 new cases of FASD in Canada each year. Positive maternal self- reports of heavy alcohol use were tenfold lower (0.24%). Use of meconium FAEE revealed tenfold more cases of heavy exposure to maternal drinking than did maternal reports. The use of objective measures of maternal alcohol exposure is critical in accurately estimating risks and in monitoring effective prevention of FASD.

## Introduction

Alcohol consumption beyond safe limits by young women is increasing^1^ and among Canadian female alcohol users, 15.9% report chronically exceeding low-risk drinking guidelines of 10 drinks per week^1^. World wide, alcohol dependence in women varies between 0 and 5.7%. and approximately 30% of pregnant women consume alcohol during pregnancy^2^. The majority of these women spontaneously discontinue consuming alcohol during their pregnancies, typically upon pregnancy recognition^1,3^. In Canada, 14% of women reported using alcohol during their last pregnancy and 5.8% report consuming alcohol during their current pregnancy^3^. In the United States, approximately 1 in 8 women report consuming alcohol during pregnancy^4^.

Heavy alcohol consumption during pregnancy is associated with increased risks of preterm delivery, spontaneous abortion, stillbirth and reduced birth weight^5,6^. Fetal alcohol spectrum disorder (FASD) is the umbrella term describing the range of fetal alcohol effects and is the leading preventable cause of mental impairment^7^. The current prevalence rates of FASD in young school-aged children in the United States has been estimated between 2–5%^8^.

Due to the range of symptoms and deficits seen in children with FASD diagnosis can be extremely challenging. The pathognomonic pattern of facial dysmorphology includes short palpebral fissures, thin upper lip and flat philtrum^9^. The highest risk of giving birth to a child with FASD occurs for mothers that report drinking during all trimesters, with the risk decreasing with earlier abstention from drinking during pregnancy^10^. It is estimated that the prevalence of FASD is 40-50% among heavily exposed neonates^11^.

Children with FASD are at an increased risk for delayed gross and fine motor skills^12^. Behavioural problems associated with FASD include attention deficit hyperactivity disorder (ADHD), conduct disorder, maladaptive and antisocial behaviour^13^. Through numerous studies, all domains of neuro-cognition have been found to be adversely affected by prenatal alcohol exposure including language, visuospatial functioning, verbal memory and learning^14^.

These behavioural deficits contribute to a series of secondary disabilities in FASD^15^ with 60% lifetime prevalence of trouble with the law^16^, inappropriate sexual behaviour, and mental health disorders^13^.

Children with FASD have significantly lower quality of life and health outcomes later on in life when compared to the general population^17^. It has been estimated that it costs the health care system and economy of Canada approximately $14000 per year for each child with FASD, totaling over $300 million for all individuals aged 1 to 21 with FASD^18^.

Most studies reporting on gestational use of alcohol rely on maternal self-reports, which are grossly inaccurate due to maternal guilt, shame and fear of losing custody of the child^19–22^. This has led to the search for biological markers of fetal alcohol exposure. Fatty acid ethyl esters (FAEEs) are non-oxidative metabolites of ethanol, formed through the esterification of ethanol with endogenous fatty acids or fatty acyl-CoA^23^. Since the discovery by Bearer that FAEE are excellent biomarkers for fetal alcohol exposure to alcohol^24^, several studies looking at baseline FAEE levels, infants born to women who did not drink alcohol during pregnancy had low meconium FAEE levels^25^. Because the body produces some ethanol during normal metabolism, FAEEs are detected in nondrinking individuals, thus requiring a clear cut-off value specific to the matrix for differentiation between heavy drinking and nondrinking individuals^25,26^. Because meconium is produced in the second and third trimesters of pregnancy, positive FAEE levels indicate late drinking, long after the woman became aware of her pregnancy; hence it is an important marker of alcohol dependence.

The objective of the present study was to estimate the prevalence of heavy fetal alcohol exposure in Canada through the analysis of FAEEs in meconium samples collected in multiple study sites across the nation.

## Subjects and Methods

### Sample and Data Collection

This study was conducted in collaboration with the Maternal-Infant Research on Environmental Chemicals (MIREC) Study Group, which has been previously described in detail^27^. The MIREC study aimed to assess the role environmental chemicals may play in the health of pregnant women and their children. The key environmental chemicals that the MIREC group was studying originally included heavy metals, phthalates, brominated flame retardants and bisphenol A^27^.

Ten cities across Canada were included in this study (Vancouver, BC; Edmonton, AB; Winnipeg, MB; Sudbury, ON; Toronto, ON; Hamilton, ON; Kingston, ON; Ottawa, ON; Montreal, QC; and Halifax, NS) recruiting a total of 2,000 volunteering women attending prenatal clinics during the first trimester of pregnancy at the participating sites between 2008 and 2011. Eligibility criteria for enrollment into the MIREC study included: age 18 years or older; <14 weeks gestation; ability to communicate in English or French; plan to deliver at a local hospital; and willingness to provide a cord blood sample. Women were excluded from the study if they had known fetal chromosomal abnormalities or major malformations in the current pregnancy, and/or a history of medical complications including epilepsy, hepatitis, cancer, hematological disorder, threatened spontaneous abortion, illicit drug use, or disease of major organs (heart, kidney, liver, lung).

Upon enrollment into the MIREC study, women were contacted in each trimester, at delivery, and up to 10 weeks post-delivery. For the purpose of the present study we used a portion of the meconium sample obtained post-delivery after the main analyses of MIREC were conducted. The meconium samples were collected from the diapers by an applicator, into a testube and kept frozen at minus 4 centigrade till transported to the Biobank. These samples were kept at the Biobank of the Institut National de Santé Publique du Québec (INSPQ). Trained research staff administered questionnaires during each trimester and conducted post-delivery interviews. All participant data were de-identified and each participant was assigned a coded ID number. Remaining bio specimens were catalogued in a Biobank at the Institut National de Santé Publique du Québec (INSPQ) when proper informed consent was obtained from participating women.

Approximately 500 mg of meconium was apportioned from each sample and transported to our laboratory at The Hospital for Sick Children (HSC) in Toronto. Meconium samples were stored in −80 °C until analysis.

The study was approved by the research ethics committees of The Hospital for Sick Children, Toronto, Saint Justine Hospital, Montreal, Health Canada, Ottawa, and MIREC. All methods were performed in accordance with the relevant guidelines and regulations.

### Sample Analysis

For meconium analysis of FAEEs, a previously published method established in our laboratory was used, comprised of a liquid-liquid extraction followed by headspace solid-phase micro-extraction (HS-SPME), coupled with gas chromatography-mass spectrometry^28^.

Briefly, calibration standards and QC samples were prepared using a FAEE mix (10 µg/mL) and a deuterated (d5) FAEE mix (10 µg/mL) of the 4 FAEEs measured (ethyl palmitate, ethyl oleate, ethyl linoleate and ethyl stearate). Extracted samples were then analyzed using headspace solid-phase micro-extraction coupled with gas chromatography-mass spectrometry (SPME GC-MS). The limits of detection (LOD) and quantification (LOQ) of each individual FAEE and the total cumulative sum are listed in Table 1. The cut-off of 2 nmol/g, established by Chan *et al*.^24^ was used to determine positivity. This cutoff value was defined in a large clinical study, in which we measured meconium FAEE levels among very religious Arab and Jewish women who do not consume any ancohol. The cutoff of 2 nmol/g was the highest level measured in meconium of non drinkers. Hence, any level above this threshold is assumed to represent external albohol consumed by the mother^24^. The inter-day and intra-day variability was 6.51% and 5.41%, and 6.45% and 5.16%, for low and high QC samples respectively. If a transitional sample (*i*.*e*., the sample was a mixture of meconium and stool) was found to be positive, it was deemed inconclusive to avoid the risk of a false positive result^28^. Transitional samples were identified either through visual inspection of the sample or by the characteristic low peak area counts of the d5-FAEE analytes.

**Table 1:** Limits of detection and quantification for the 4 FAEEs analyzed in meconium by GC-MS.

Individual FAEE	LOD (ng/vial)	LOQ (ng/vial)	
**Limits of Detection & Limits of Quantification**			
Palmitic	5.902	17.706	
Linoleic	5.961	17.882	
Oleate	3.839	11.517	
Stearate	3.127	9.380	
Total Cumulative Sum LOD* LOQ* Upper limit of quantification*			
FAEE	0.125	0.400	26

*Nmol/g meconium.

### Data Analysis

**Figure 1:**
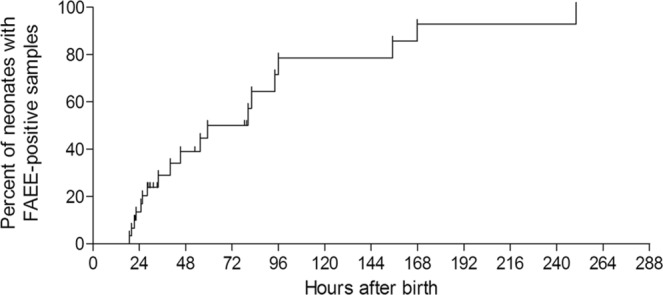
Rates of positive meconium FAEE test as a function of time after birth, among neonates that started with negative tests at baseline (from ref.^29^).

**Figure 2:**
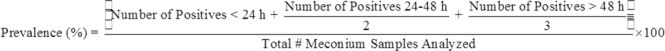
The equation to calculate the prevalence of positive meconium FAEE as a function of the postnatal hours of meconium secretion(based on the data from Fig. 1).

Prevalence of heavy *in utero* exposure to alcohol was calculated from the meconium FAEE results for the entire study population. To account for time of meconium collection after birth, data from our previous research were utilized to adjust for the elevated risk of false positive results, as described earlier^29^: In our previous research, after serial meconium collection from the same neonates, the false positive rate was approximately 20%, 40% and 60% after 24, 48 and 72 hours, respectively (Fig. 1, ref.^29^). The equation in Fig. 2 was used to calculate the true prevalence rates of positive meconium FAEE (Fig. 2):

Meconium FAEE analysis results were compared to maternal alcohol consumption self-report questionnaires from the third trimester (between 32 and 35 weeks gestation)

## Results

Overall, out of a total of 2000 women approached, 1436 meconium samples were available from the MIREC Biobank, with 1315 eligible for FAEE analysis and 125 either not having sufficient quantity (NSQ) for analysis or were identified to be postnatal stool. By utilizing Equation 1, the mean prevalence of heavy fetal alcohol exposure was 1.16%. Including only the samples collected within the first 24 postnatal hours, the prevalence rate of positive meconium test was 2.40% (Table 2).

**Table 2:** FAEE meconium results and estimated overall prevalence of heavy fetal alcohol exposure in Canada.

Meconium Result	Meconium Collection		Time after birth		Total
	<24 h	24-48 h	>48 h	N/A	
Positive	4	12	16	0	32
Negative	157	478	480	7	1122
Inconclusive	6	63	90	2	161
				TOTAL	1315
				CorrectedPrevalence (equation 1)	1.16%

Comparing the overall meconium results to the overall maternal self-report generated from the 32–35 week gestation questionnaire, the majority of women reported no alcohol consumption during pregnancy, with 32% reporting social level drinking (less than 2 standard drinks per week). There was no relationship between level of alcohol consumption reported by the mothers and FAEE in meconium. A small percentage, 0.24%, reported above social level drinking, defined as more than 2 standard drinks per week. This rate was tenfold lower than the positive rate of meconium FAEE (2.4%).

## Discussion

To our knowledge, this is the first study of its kind to estimate a national prevalence of heavy *in utero* alcohol exposure through a biological marker using a multi-center cohort which spans across Canada. Through FAEE meconium analysis, it can now be estimated that the rate of heavy *in utero* alcohol exposure in Canada ranges between 1.16 and 2.40%. When comparing the self-reported alcohol consumption and the respective meconium results, the meconium analysis identified a tenfold larger proportion of heavy alcohol consumption during pregnancy (0.234% vs. 2.4%). This speaks to the limitations of self-report that have been documented numerous times^30^.

The upper part of this estimated range is similar to previously reported prevalence rates in studies conducted in Canada^31–33^. This prevalence rate should be considered as an estimate of the true rate of heavy *in utero* alcohol exposure. As seen in a previous study focusing on a high-risk obstetric unit, the prevalence rate in this type of population is significantly higher than the general population^32^. As the MIREC study cohort has been shown to have a higher level of education and annual household income than the Canadian average, due to the voluntary nature of participation in the study, this estimate most likely refers to a higher SES portion of the Canadian population.

Approximately 32% of women reported alcohol consumption in the past 3 months in the 32–35-week gestation questionnaire. While the majority of the alcohol consumption was reported to be at a social level, this further supports previous findings that a substantial minority of women continue drinking throughout their pregnancy despite warnings and proven long-term risks^34^. There was no relationship between level of alcohol consumption reported by the mothers and FAEE in meconium. This agrees and corroborates previous reports that women’s reporting on drinking in pregnancy is not accurate due to fears, shame and guilt, with strong element of under reporting^17^.

Increasing levels of FAEEs in meconium have been associated with poorer psychomotor and mental outcomes in children up to 2yr. of age^35^. In parallel, increasing doses of alcohol during pregnancy have been found to result in more pronounced and prominent effects on the fetus^36^. The relevance of these results to other countries was recently documented in a study from Scotland using meconium FAEEs, documenting that at least 15% of women in an inner-city maternity unit in the west of Scotland were consuming significant quantities of alcohol during latter pregnancy^37^.

Several potential limitations of the study need to be acknowledged. While the multicentre nature of this study helps provide a more accurate prevalence rate by including a larger population, these results may not be generalizable to the population of Canada, but rather to a higher SES stratum and people residing in or near large cities. This may mean that the true prevalence of positive FAEE may be higher than the rate we observed, as inverse relation between SES and alcohol consumption in pregnancy has been described^38^, but refuted in other recent studies^39^. With respect to meconium, the positive cut-off of cumulative FAEE levels (ethyl palmitate, oleate, stearate, linoleate) was established at 2nmol/g (~600 ng/g)^23^, differentiating between heavy drinkers vs. non-drinkers / social drinkers^20^. Timing of light alcohol exposure, either during the second or third trimesters of pregnancy, did not affect FAEE levels in meconium either^22^. Other cut-offs have been proposed and used in FAEE analysis. It was suggested that a total FAEE concentration (ethyl palmitate, linoleate, oleate, stearate, palmitoleate, linoleate) positive cut-off of 10,000 ng/g (approximately 33 nmol/g) should be used; however, this cut-off may only identify very significant alcohol exposure. Other cut-offs proposed involve up to 9 FAEE species and ranges from 200 ng/g to 600 ng/g. The sensitivity and specificity of these cut-offs vary between 52%–100% and 45.1%–98.4% respectively^37,40^.

Another potential limitation is that meconium is produced during the second and third trimesters, and thus positive FAEE levels indicate late drinking, long after the woman became aware of her pregnancy; hence it is an important marker of alcohol dependence. While this biomarker misses the first trimester, the more sensitive period of embryogenesis, most alcohol-dependent women who drink in the third trimester also had drank in early pregnancy.

A potential limitation of FAEE meconium testing is that samples excreted later in the postpartum period have higher levels of FAEEs than earlier collected samples for the same infant due to de novo production of alcohol from carbohydrates in the meconium. This could lead to false-positive FAEE results, which was adjusted for in the present study^29^.Given the rising false positive rates that are seen in the days postpartum due to de novo carbohydrate metabolism, the only way to implement this technique for patient analysis is to conduct the test on first- day meconium. Presently, the potential effects of the woman’s or fetal demographics and medical history on FAEE levels have not been studied. In animal studies, the activity of cytochrome P450 CYP2E1 was shown to be inversely related to meconium FAEE concentrations^41^.

The major issue in calculating sensitivity and specificity of meconium FAEE as a marker of alcohol intake in humans, is that one can never verify maternal alcohol consumption. However, in animal models, where the amount of ethanol given to the pregnant animal is known, we and others have shown very high values of sensitivity and specificity. In an ovine model Littner has shown ethyl oleate concentrations above 131 ng/g has a sensitivity of 89% and specificity of 100% for ethnol exposure^42^.We have shown sensitivity and specificity of 93.3% for cumulative meconium levels above 0.03nmol/g in an ovine model with maternal dose of 0.75 g/kg /d of maternal body weight^43^.

Another recently described potential biological marker of fetal alcohol exposure is the measurement of a different metabolite of alcohol, phosphatidyl-ethanol through Gathrie cards^39^. However, its clinical utility has not been yet demonstrated.

Overall, the present study found an estimated rate of heavy *in utero* alcohol exposure in Canada ranging between 1.16 and 2.40%. This prevalence rate provides a better indication of what the current trends of alcohol consumption during pregnancy are in Canada. The self-reported alcohol consumption data showed a tenfold lower prevalence, reflecting the inherent problems in self reports of gestational alcohol consumptions due to guilt, shame and fears of prosecution and losing custody of the child. Using the accepted 50% risk of FASD following heavy alcohol exposure in pregnancy^40^, the most conservative prevalence rate calculated in the present study, 1.16%, is the equivalent of approximately 1800 new cases of FASD each year in Canada. At a cost of over 1 million dollars per new case, this translates to 2 billion dollars a year, on top of the immense human suffering and poor quality of life.

With FASD being the most prevalent preventable cause of mental impairment, the use of objective biomarkers of maternal alcohol exposure such as meconium FAEE will be critical to accurately identify the size of the risk, and to follow up implementation of prevention strategies, such as harm reduction. Beyond quantifying the true extent of the problem within populations to encourage better investment in harm minimisation strategies, this can be a promising early intervention strategy that could be employed for identified affected children that could minimise the future suffering for them and their associated health care costs. We have initiated an intervention program for newborns identified with positive FAEE, which includes followup by child workers and psychologists, to tailor appropriate interventions when the first signs of difficulties arise^44^.
